# Discovery of novel secondary metabolites encoded in actinomycete genomes through coculture

**DOI:** 10.1093/jimb/kuaa001

**Published:** 2021-01-25

**Authors:** Ji Hun Kim, Namil Lee, Soonkyu Hwang, Woori Kim, Yongjae Lee, Suhyung Cho, Bernhard O Palsson, Byung-Kwan Cho

**Affiliations:** Department of Biological Sciences and KI for the BioCentury, Korea Advanced Institute of Science and Technology, Daejeon 34141, Republic of Korea; Department of Biological Sciences and KI for the BioCentury, Korea Advanced Institute of Science and Technology, Daejeon 34141, Republic of Korea; Department of Biological Sciences and KI for the BioCentury, Korea Advanced Institute of Science and Technology, Daejeon 34141, Republic of Korea; Department of Biological Sciences and KI for the BioCentury, Korea Advanced Institute of Science and Technology, Daejeon 34141, Republic of Korea; Department of Biological Sciences and KI for the BioCentury, Korea Advanced Institute of Science and Technology, Daejeon 34141, Republic of Korea; Department of Biological Sciences and KI for the BioCentury, Korea Advanced Institute of Science and Technology, Daejeon 34141, Republic of Korea; Department of Bioengineering, University of California, San Diego, La Jolla, CA 92093, USA; Department of Pediatrics, University of California, San Diego, La Jolla, CA 92093, USA; Novo Nordisk Foundation Center for Biosustainability, Technical University of Denmark, 2800 Lyngby, Denmark; Department of Biological Sciences and KI for the BioCentury, Korea Advanced Institute of Science and Technology, Daejeon 34141, Republic of Korea; Intelligent Synthetic Biology Center, Daejeon 34141, Republic of Korea

**Keywords:** Actinomycetes, *Streptomyces*, Coculture, Secondary metabolite

## Abstract

Actinomycetes are a rich source of bioactive natural products important for novel drug leads. Recent genome mining approaches have revealed an enormous number of secondary metabolite biosynthetic gene clusters (smBGCs) in actinomycetes. However, under standard laboratory culture conditions, many smBGCs are silent or cryptic. To activate these dormant smBGCs, several approaches, including culture-based or genetic engineering-based strategies, have been developed. Above all, coculture is a promising approach to induce novel secondary metabolite production from actinomycetes by mimicking an ecological habitat where cryptic smBGCs may be activated. In this review, we introduce coculture studies that aim to expand the chemical diversity of actinomycetes, by categorizing the cases by the type of coculture partner. Furthermore, we discuss the current challenges that need to be overcome to support the elicitation of novel bioactive compounds from actinomycetes.

## Introduction

Natural products are organic compounds produced by living organisms mainly in the form of secondary metabolites, most of which have therapeutic bioactivity, including antimicrobial, antifungal, and anticancer (Harvey, 2008). The representative sources of these bioactive secondary metabolites are Gram-positive soil-living bacteria actinomycetes, particularly *Streptomyces*, whose products comprise approximately 70% of commercially available antibiotics (Nett et al., 2009). From the 1950s to 1970s, the golden period of antibiotic discovery, a number of compounds produced by *Streptomyces* strains were explored and utilized to deal with infectious diseases (Aminov, 2010; Procopio et al., 2012). However, after two decades of success, antibiotic discovery became depressed owing to the continuously increasing rediscovery rate of known chemical entities, while pathogenic microbes gradually cultivated antimicrobial resistance to the latest generation of antibiotics (Koehn & Carter, 2005; Ventola, 2015). Even worse, currently, the emergence of multidrug-resistant pathogens such as “ESKAPEE” (*Enterococcus faecium, Staphylococcus aureus, Klebsiella pneumoniae, Acinetobacter baumannii, Pseudomonas aeruginosa, Enterobacter* species, and *Escherichia coli*) has triggered an urgent need for new and improved antimicrobial drugs (Boucher et al., 2009; Pendleton et al., 2013; Rice, 2008; Tacconelli et al., 2018).

Recent advances in high-throughput genome sequencing techniques and *in silico* genome mining tools have elucidated that actinomycetes, especially *Streptomyces*, possess a tremendous number of unexplored secondary metabolite biosynthetic gene clusters (smBGCs), indicating that the biosynthetic capability of *Streptomyces* has been underestimated (Craney et al., 2013; Lee et al., 2020b). For example, the genome mining of *Streptomyces griseus*, a well-known producer of the first aminoglycoside antibiotic streptomycin, identified 34 smBGCs in the genome, which include 28 putative smBGCs in addition to the previously characterized 6 smBGCs (Ohnishi et al., 2008). Considering that 1,110 *Streptomyces* strains possess approximately 40 smBGCs on average (Belknap et al., 2020) and that other actinomycete families such as *Pseudonocardiales, Streptosporanqineae, Micromonosporaceae*, and *Corynebacteriales* have 19.8, 15.0, 13.3, and 8.4 smBGCs per genome, respectively (Doroghazi et al., 2014), the genetic potential of actinomycetes has not been fully utilized because most of the smBGCs are apparently silent (cryptic) under laboratory pure culture conditions. Secondary metabolites are involved in inter- or intraspecies interactions in the natural habitat of the producer, but they are not essential for cell growth. Moreover, secondary metabolites are assembled by mega-enzyme complexes, the expression of which requires a large amount of energy and resources. Thus, the expression of smBGCs is inhibited until the action of specific environmental stimuli, such as microbial competition and physical stresses from the natural habitat.

To overcome this limitation, a variety of strategies have been developed and applied to activate the silent or poorly expressed smBGCs of actinomycetes. These approaches also provided useful information for understanding the regulatory mechanisms related to secondary metabolism. The culture-based method “OSMAC” (one strain many compounds) is one of the basic and simple ways to activate silent smBGCs (Bode et al., 2002). By changing culture conditions, including media composition (e.g., nutrient contents and chemical elicitors) (Chen et al., 2000; Kawai et al., 2007; Pettit, 2011; Tanaka et al., 2010) or physical parameters (e.g., temperature, pH, osmotic stress, and salinity) (Bode et al., 2002), a single strain can be induced to produce various molecules. Genetic engineering-based smBGC activation methods categorized into targeted (e.g., promoter exchange, heterologous expression, and cluster-situated regulator engineering) (Laureti et al., 2011; Luo et al., 2013; Zhang et al., 2017) and non-targeted (e.g., ribosome engineering and global regulator engineering) approaches are also widely used to induce substantial changes in the secondary metabolism of actinomycetes (Gao et al., 2012; Hosaka et al., 2009).

In addition to the aforementioned conventional strategies, coculture of different species is also effective in awakening silent smBGCs. Compared to the conventional strategies, coculture has the advantage of simplicity in that there is no need of prior knowledge of smBGCs or genetic engineering tools (Reen et al., 2015; Romano et al., 2018). Furthermore, coculture not only mimics ecological stresses like nutrient depletion during interspecies competition (Patin et al., 2018; van Bergeijk et al., 2020), but also enables real-time monitoring of secondary metabolite bioactivities toward the participants of coculture via the analysis of morphological changes or cell density (Wu et al., 2010). Under these conditions, several ideal combinations of the producer and partner (inducer) have been identified, which efficiently induce production of novel secondary metabolites, including antibiotics, antifungals, anticancers, and siderophores. However, owing to the chemical and molecular complexity of microbial interaction, the precise underlying mechanisms of the interaction are remarkably unexplored.

**Fig. 1.**
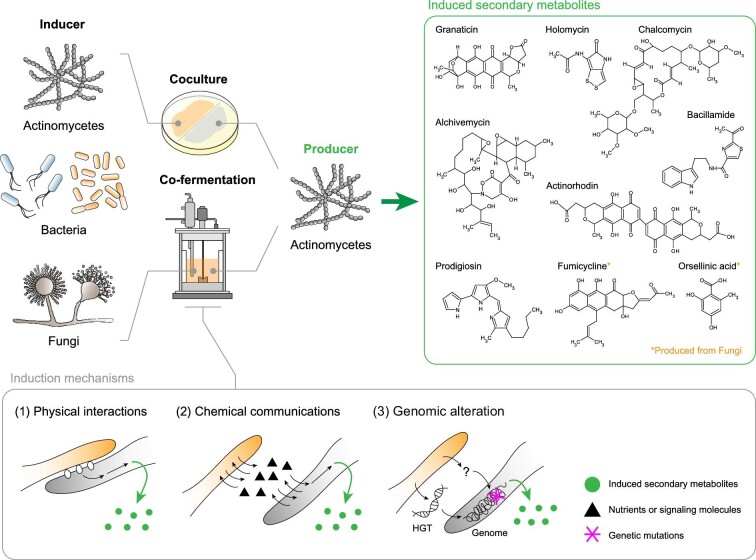
Overview of expanding chemical diversity of actinomycetes via coculture. HGT, horizontal gene transfer.

In this review, we briefly introduce the conventional strategies to awaken the silent smBGCs, and thereafter, focus on the coculture approach for unlocking the secondary metabolite production potential of actinomycetes. Coculture approaches are categorized into three sections depending on the coculture partners (Fig. 1): (i) actinomycetes–actinomycetes, (ii) actinomycetes–non-actinomycetes bacteria, and (iii) actinomycetes–fungi. The difference between bacteria and fungi as participants in coculture is presented from the perspective of induced secondary metabolites, bioactivity of secondary metabolites, and producer–inducer relationship. Finally, we highlight the future challenges of increasing the chemical diversity of actinomycetes using coculture.

## Conventional Strategies for Awakening Silent smBGCs

Genome sequencing and genome mining approaches have revealed numerous potential smBGCs from actinomycetes. However, most of them are inactive under laboratory culture conditions and only subsets of these smBGCs are produced. To activate these silent smBGCs, various strategies have been developed that could be categorized into (i) culture-based strategies and (ii) genetic engineering-based strategies.

In the ecological habitat of secondary metabolite producers, biotic stresses (e.g., nutrient competition with nearby microbes) and abiotic stresses (e.g., acidity, drought, temperature, and salinity) are prevalent, which stimulate the production of various secondary metabolites (Cihak et al., 2017). In this respect, altering the culture conditions of actinomycetes is a simple and basic approach for unlocking cryptic smBGCs, which has been labeled the OSMAC approach (Bode et al., 2002). Secondary metabolite production is usually initiated when the cell growth slows down, indicating that exhaustion of a nutrient is a major key for awakening the silent smBGCs (Bibb, 2005). Therefore, changing nutrient regimes like carbon, nitrogen, sulfur, phosphorus, or trace element sources has been implemented for the secondary metabolite production from actinomycetes. Carbon source, in particular, is one of the main factors that controls secondary metabolite production (Sanchez et al., 2010). Rapidly used or preferred carbon sources, such as glucose, are known to repress the biosynthesis of various secondary metabolites in actinomycetes (i.e., carbon catabolite repression) (Bhatnagar et al., 1988; Sankaran & Pogell, 1975); thus, decreasing or altering the repressing carbon source could increase or induce inactivated secondary metabolite production. For example, actinorhodin production by *Streptomyces lividans* is inhibited when glucose is used as a carbon source, whereas inhibition is relieved when glucose is replaced with glycerol (Kim et al., 2001). In addition, modifying physical culture conditions, including temperature, salt concentration, or pH, also has a dramatic effect on the hierarchical regulatory network of actinomycetes and induces the production of novel secondary metabolites. For example, recently 18 types of thermotolerant actinomycetes were cultured between 30 and 45°C, and secondary metabolite production was compared. As a result, it was found that 131 secondary metabolites were produced when the actinomycetes were cultured at high temperature (Saito et al., 2020). Production of several secondary metabolites was induced in order to deal with the changed physical culture condition, as in the case of *Nocardiopsis gilva* YIM 90087 that accumulates ectoine and hydroxyectoine under salt stress conditions in order to regulate osmotic pressure (Han et al., 2018).

Genetic engineering-based strategies are promising for activating either (i) targeted or (ii) non-targeted smBGCs, if genome sequences and genetic manipulation tools for target actinomycetes are available. First, in the case of targeted smBGC activation, by altering genetic components, such as promoters of smBGC-encoded genes, expression of silent smBGCs could be stimulated. Recently, CRISPR/Cas9 systems have been applied to several *Streptomyces* species, enabling insertion of a strong and constitutive promoter in the upstream of the core biosynthetic genes or positive regulatory genes encoded in the target smBGC (Cobb et al., 2015; Huang et al., 2015; Zhang et al., 2017). For example, activation of pentangular type II polyketide BGC of *Streptomyces viridochromogenes* via CRISPR/Cas9-mediated promoter exchange of the main biosynthetic operon resulted in the production of a novel pigmented compound (Zhang et al., 2017). Meanwhile, targeted smBGC awakening in native hosts is often hampered by endogenous complex regulatory systems; thus, in many cases, smBGCs of interest are expressed in heterologous hosts to bypass the original regulatory systems. For instance, a PKS–NRPS-type BGC of *S. griseus* containing nine domains of biosynthetic mega-enzyme was reconstructed and heterologously expressed in *S. lividans*, resulting in the production of three novel tetramic acid-containing macrolactams (Luo et al., 2013). Additionally, non-targeted smBGC activation relies on reshaping the global transcriptome or translatome via genetic engineering, followed by analyzing the change in produced secondary metabolite pools. A representative method involves altering the expression of pleiotropic transcriptional regulators. For instance, overexpression of cyclic AMP receptor protein (Crp), which is a transcription regulator involved in diverse cellular processes, enhanced secondary metabolite production ability of various *Streptomyces* species, including *S. coelicolor* (Gao et al., 2012). In addition, introducing mutations in RNA polymerase or ribosomal proteins to change transcriptional or translational activity, respectively, led 66 strains out of 353 soil-isolated actinomycetes to acquire an antibacterial-producing ability (Hosaka et al., 2009).

## Coculture of Actinomycetes

Coculture is another effective culture-based strategy for discovering novel bioactive secondary metabolites from microorganisms by mimicking the environmental habitat where microbes continuously interact with nearby residents. It is defined as “coculture or co-cultivation” when performed on solid media, such as Petri dishes or a solid support system, and called “mixed fermentation” when performed in liquid media, such as co-fermentation, transwell, microfluidic, or droplet culture systems (Tan et al., 2019). Compared to conventional strategies, coculture offers complex and unpredictable stimuli over the sole nutrient or physical condition changes, allowing microbes to produce various novel secondary metabolites, which are not observed in pure culture conditions (Abdelmohsen et al., 2015). Also, coculture enables the real-time bioactivity screening of newly induced secondary metabolites when producers are cocultured with target pathogens. Furthermore, the coculture method is beneficial not only for awakening novel secondary metabolites but also for comprehending microbial interactions related to complex regulations of secondary metabolite production. In this context, coculture methods have been intensively applied to bacteria and fungi, especially to actinomycetes (Abdelmohsen et al., 2015; Yu et al., 2019). In this section, various actinomycete coculture studies are classified into three categories, depending on the type of coculture partner, as follows: (i) actinomycetes–actinomycetes, (ii) actinomycetes–non-actinomycetes bacteria, and (iii) actinomycetes–fungi.

### Actinomycetes Coculture With Actinomycetes

#### *Streptomyces* coculture with *Streptomyces*

More than 3,000 species of *Streptomyces* reside together in their ecological habitats and numerous interspecies interactions exist within them (Christova et al., 1995); therefore, many attempts have been made to coculture different *Streptomyces* species to expand the chemical diversity of *Streptomyces* (Table 1). For example, coculture of 76 *Streptomyces* species revealed that production of various antibiotics or sporulation was induced in 72 combinations (Ueda et al., 2000). Interspecies interaction mediated by diffusible substrates (e.g., γ-butyrolactones [GBLs] and secondary metabolites themselves) is regarded as a general factor triggering the secondary metabolism during *Streptomyces*–*Streptomyces* coculture. Especially, GBLs (e.g., A-factor, virginiae butanolides, and IM-2) are well-known and widely distributed signaling molecules involved in communications of *Streptomyces* species (Niu et al., 2016). GBLs produced from various *Streptomyces* species including *S. viridochromogenes, S. bikiniensis*, and *S. cyaneofuscatus* induced antibiotic production, cellular differentiation, and aerial mycelium formation of *S. griseus*, as A-factor, the GBL of *S. griseus*, did (Grafe et al., 1983; Hara & Beppu, 1982; Horinouchi & Beppu, 1992; Khokhlov et al., 1973; Yamada et al., 1987).

**Table 1.** Actinomycetes and Actinomycetes Coculture

Producer	Inducer	Induced compounds and bioactivity	Category (producer–inducer)	References
*Streptomyces* 76 species	*Streptomyces* 76 species	Various antibiotics from 72 combinations	S–S	Ueda et al. (2000)
*Streptomyces* 33 isolates	*Streptomyces* 33 isolates	Various antibiotics from 31 combinations	S–S	Ueda et al. (2000)
*Streptomyces griseus* st-21-2	*Streptomyces tanashiensis* IAM0016	Desferrioxamine E (siderophore)	S–S	Yamanaka et al. (2005)
*Streptomyces tanashiensis* IAM0016	*Streptomyces griseus* st-21-2	Unknown antibiotics	S–S	Yamanaka et al. (2005)
*Streptomyces coelicolor* M145	*Streptomyces* sp. E14	Four acyl-desferrioxamine derivatives (siderophore)	S–S	Traxler et al. (2013)
*Streptomyces coelicolor* M145	*Streptomyces* sp. SPB74	γ-Actinorhodin (antibiotics)Prodiginine (antibiotics)Acyl-desferrioxamines (siderophore)	S–S	Traxler et al. (2013)
*Streptomyces coelicolor* M145	*Streptomyces viridochromogenes*	Actinorhodin (antibiotics)Prodiginine (antibiotics)Coelichelin (siderophore) Desferrioxamine E (siderophore)	S–S	Traxler et al. (2013)
*Streptomyces coelicolor* M145	*Streptomyces albus* J1074	Actinorhodin (antibiotics)Prodiginine (antibiotics) Desferrioxamines B and E (siderophore)	S–S	Traxler et al. (2013)
*Streptomyces* strains 574, 001, 023, and 555	*Streptomyces* strain 153	Unknown antibiotics	S–S	Amano et al. (2010)
*Streptomyces lividans* TK-23	*Tsukamurella pulmonis* TP-B0596*Rhodococcus* sp*.Corynebacterium* sp*.Nocardia* sp*.Dietzia* sp*.Gordonia* sp*.Mycobacterium* sp*.Williamsia* sp.	Actinorhodin (antibiotics)Undecylprodigiosin (antibiotics)	S– MACB	Onaka et al. (2011)
*Streptomyces endus* S-522	*Tsukamurella pulmonis* TP-B0596*Corynebacterium glutamicum*	Alchivemycins A and B (antibiotics)	S–MACB	Onaka et al. (2011)
*Streptomyces cinnamoneus* NBRC 13823	*Tsukamurella pulmonis* TP-B0596	BE-13793C (cytotoxicity)Arcyriaflavin E (cytotoxicity)Arcyriaflavin A	S–MACB	Hoshino et al. (2015c)
*Streptomyces* sp. CJ-5	*Tsukamurella pulmonis* TP-B0596	Chojalactones A–C (cytotoxicity)	S–MACB	Hoshino et al. (2015b)
*Streptomyces* sp. NZ-6	*Tsukamurella pulmonis* TP-B0596	Niizalactams A–C	S–MACB	Hoshino et al. (2015a)
*Streptomyces lividans* TK-23	*Tsukamurella pulmonis*	Prodiginine (antibiotics)	S–MACB	Onaka et al. (2015)
*Streptomyces nigrescens* HEK616	*Tsukamurella pulmonis* TP-B0596*Corynebacterium glutamicum*	5-Alkyl-1,2,3,4-tetrahydroquinoline (antifungal)	S–MACB	Sugiyama et al. (2015)
*Streptomyces nigrescens* HEK616	*Tsukamurella pulmonis* TP-B0596	Streptoaminals (antifungal)	S–MACB	Sugiyama et al. (2016)
*Streptomyces tendae* KMC006	*Gordonia* sp. KMC005	Gordonic acid (antibiotics)	S–MACB	Park et al. (2017)
*Nocardiopsis* sp. FU40	*Rhodococcus wratislaviensis*	Ciromicins A and B (cytotoxicity)	A–MACB	Derewacz et al. (2015)
12 *Micromonosporaceae*	*Mycobacterium* sp. WMMA-183*Rhodococcus* sp. WMMA-185	Unknown antibiotics	A–MACB	Adnani et al. (2015)
*Micromonospora* sp. WMMB235	*Rhodococcus* sp. WMMA185	Keyicin (antibiotics)	A–MACB	Adnani et al. (2017)
*Micromonospora wenchangensis* HEK797	*Tsukamurella pulmonis* TP-B0596	Dracolactams A and B	A–MACB	Hoshino et al. (2017)
*Catenuloplanes* sp. RD067331	*Tsukamurella pulmonis* TP-B0596	Catenulobactin ACatenulobactin B (siderophore and cytotoxicity)	A–MACB	Hoshino et al. (2018a)
*Actinosynnema mirum* NBRC 14064	*Tsukamurella pulmonis* TP-B0596	Mirilactams C–E	A–MACB	Hoshino et al. (2018b)
*Pseudonocardiales Umezawaea* sp. RD066910	*Tsukamurella pulmonis* TP-B0596	Umezawamides A and B (cytotoxicity)	A–MACB	Hoshino et al. (2018c)
*Nocardiopsis* sp. RV163 (producer unknown)	*Actinokineospora* sp. EG49	*N*-(2-Hydroxyphenyl)-acetamide1,6-Dihydroxyphenazine5a,6,11a,12-Tetrahydro-5a,11a-dimethyl[1,4]benzoxazino[3,2b][1,4]benzoxazine (antibiotics and antitrypanosomal)	A–A	Dashti et al. (2014)
*Streptomyces coelicolor* M145	*Amycolatopsis* sp. AA4	γ-Actinorhodin (antibiotics)Prodiginine (antibiotics)Four acyl-desferrioxamine derivatives (siderophore)Amychelin from *S. coelicolor* (siderophore)	S–A	Traxler et al. (2012, 2013)
*Rhodococcus fascians* 307CO	*Streptomyces padanus*	Rhodostreptomycins A and B (antibiotics)	MACB–S	Kurosawa et al. (2008)

S: *Streptomyces*; MACB: mycolic acid-containing bacteria; A: actinomycetes.

Secondary metabolites themselves also play crucial role in promoting production of various secondary metabolites between *Streptomyces*–*Streptomyces* interactions. Among the secondary metabolites, iron-chelating compound, siderophore, is a type of secondary metabolite that stimulates secondary metabolism, such as antibiotic production or development of another nearby species (Challis & Hopwood, 2003). Desferrioxamine E, which is a siderophore produced by *S. griseus*, stimulated growth and antibiotic production of *Streptomyces tanashiensis* (Yamanaka et al., 2005). In addition, siderophores made by four different *Streptomyces* species and *Amycolatopsis* sp. AA4 induced production of γ-actinorhodin, prodiginine, or 12 different desferrioxamines from *S. coelicolor* (Traxler et al., 2013). While iron competition with neighboring strains is suspected to be the reason for increased secondary metabolite production of *S. coelicolor*, the underlying mechanism inducing the other secondary metabolites remains to be elucidated. Meanwhile, non-siderophore secondary metabolites were also involved in *Streptomyces* interspecies communications. For example, polyether antibiotic promomycin, produced by *Streptomyces* stain 153, induced the production of unknown antibiotics from other *Streptomyces* species. Polyether antibiotics act as ionophore, which increases K^+^ ion efflux through cell membrane by forming pores; thus, it is supposed to inhibit bacterial growth and induce the production of antibiotics. Indeed, other polyether antibiotics including salinomycin, monensin, and nigericin all promoted the antibiotic production of *Streptomyces* strain 574 (Amano et al., 2010). Taken together, *Streptomyces*–*Streptomyces* coculture examples pointed out that signaling molecules involved in interspecies interactions between *Streptomyces* species triggered production of cryptic secondary metabolites and other interaction-mediating chemicals have the potential to be utilized as cues for increasing the chemical diversity of *Streptomyces*.

#### *Streptomyces* coculture with non-*Streptomyces* actinomycetes

In addition to *Streptomyces*–*Streptomyces* coculture, intergenus interactions between *Streptomyces* and non-*Streptomyces* actinomycetes have also been exploited to activate dormant smBGCs of *Streptomyces* (Table 1). Coculturing *S. lividans* with 400 different bacteria discovered that *Tsukamurella pulmonis*, a rare actinomycete, is an effective coculture partner that activated prodiginine production by *S. lividans* (Onaka et al., 2011). In addition, several *Tsukamurella*-related actinomycetes such as *Rhodococcus, Corynebacterium, Nocardia, Dietzia, Gordonia, Mycobacterium*, and *Williamsia* showed the same effect (Onaka et al., 2011). Common characteristic of these close actinomycetes is the presence of mycolic acid in the outer layer of the cells, so they are called mycolic acid-containing bacteria (MACB). These MACB have been widely cocultured with various *Streptomyces* species, and consequently induced production of numerous secondary metabolites with a variety of bioactivity including antibacterial (e.g., alchivemycin, prodiginine, streptoaminals, and gordonic acid) (Onaka et al., 2011, 2015; Park et al., 2017; Sugiyama et al., 2016), antifungal (e.g., 5a-THQ and streptoaminals) (Sugiyama et al., 2015, 2016), and cytotoxic (e.g., BE-13793C, arcyriaflavin E, and chojalactones A–C) (Hoshino et al., 2015b). Coculturing MACB with non-*Streptomyces* actinomycetes also successfully awakened several cryptic smBGCs. For example, *Mycobacterium* sp. and *Rhodococcus* sp. induced production of several secondary metabolites from 12 out of 65 marine invertebrate-associated *Micromonosporaceae* (Adnani et al., 2015).

However, the underlying mechanism of MACB coculture is still ambiguous. Most of MACB coculture studies argued that physical cell-to-cell contact between actinomycetes and live MACB cells is required for inducing secondary metabolite production of actinomycetes, because both MACB culture extract treatment and dead MACB cell coculture were not able to induce secondary metabolite production (Onaka et al., 2011). On the contrary, keyicin production of *Micromonospora* sp. WMMB235 was still observed when only the chemical substance from MACB was treated, indicating that physical contact is not required (Adnani et al., 2017). Moreover, there is a report that horizontal gene transfer between MACB and actinomycetes induces the production of a novel secondary metabolite called rhodostreptomycin, although MACB is the producer and its partner actinomycete is the inducer in this case (Kurosawa et al., 2008). Overall, silent or poorly expressed smBGCs of actinomycetes could be induced by coculture between actinomycetes (Table 1). Interspecific signaling molecules between *Streptomyces* species, including siderophore, and intergenus communications between MACB and actinomycetes triggered production of numerous bioactive compounds. Further mechanical studies on the microbial interactions that trigger the secondary metabolism will provide valuable information to understand the regulatory network of secondary metabolism and to increase the chemical diversity of actinomycetes.

### Actinomycetes Coculture With Non-Actinomycetes Bacteria

#### Actinomycetes coculture with predatory bacteria

As actinomycetes dwell in various habitats with diverse species, they have long evolved while interacting with many coexisting bacteria (Baltz, 2008; Jose & Jebakumar, 2012; Quillet et al., 1995). Among these bacteria, several predatory groups, which feed on nearby bacterial cells in the environmental habitat, are attractive coculture partners to stimulate protective response of the actinomycetes. For example, when motile predator bacteria *Myxococcus xanthus* was cocultured with *S. coelicolor, M. xanthus* secreted lytic enzymes, which triggered abnormal hyphae formation of *S. coelicolor*, and *S. coelicolor* produced actinorhodin to repel the intrusion of the *M. xanthus* (Perez et al., 2011). Although other bacteria, including several *Bacillus* species (*B. megaterium, B. subtilis*, and *B. thuringiensis*) and *Serratia* sp., slightly induced the production of actinorhodin from *S. coelicolor, M. xanthus* was the strongest inducer, representing the potential of predatory bacteria as coculture partner (Perez et al., 2011) (Table 2).

**Table 2.** Actinomycetes and Non-Actinomycetes Bacteria

Producer	Inducer	Induced compounds and bioactivity	Category (producer–inducer)	References
*Streptomyces coelicolor* M145	*Myxococcus xanthusBacillus megateriumBacillus subtilisBacillus thuringiensisSerratia* sp.	Actinorhodin (antibiotics)	S–PRB	Perez et al. (2011)
*Streptomyces coelicolor* M145	*Myxococcus xanthus*	Actinorhodin (antibiotics)Myxochelin from *M. xanthus* (siderophore)	S–PRB	Lee et al. (2020a)
*Streptomyces* sp. PTY087I2	Methicillin-sensitive *Staphylococcus aureus* Methicillin-resistant *Staphylococcus aureusPseudomonas aeruginosa*	Granatomycin D (antibiotics)Granaticin (antibiotics)Dihydrogranaticin B	S–PAB	Sung et al. (2017)
*Streptomyces albogriseolus* B24	*Bacillus cereus*	Dentigerumycin E (anticancer)	S–PAB	Shin et al. (2018)
*Streptomyces clavuligerus* (adapted by ALE)	*Staphylococcus aureus* N315	Holomycin (antibiotics)	S–PAB	Charusanti et al. (2012)
*Streptomyces coelicolor* M145	*Staphylococcus aureus* (heat-killed cell)	Undecylprodigiosin (antibiotics, immunosuppressive, and anticancer)	S–PAB	Luti and Mavituna (2011)
*Streptomyces* sp. PTY087I2	*Bacillus subtilis*	Granatomycin D (antibiotics)Granaticin (antibiotics)Dihydrogranaticin B	S–NB	Sung et al. (2017)
*Streptomyces coelicolor*	*Bacillus subtilis* (bacillaene-deficient)	Undecylprodigiosin (antibiotics, immunosuppressive, and anticancer)	S–NB	Straight et al. (2007)
*Streptomyces coelicolor* M145	*Bacillus subtilis* (heat-killed cell)	Undecylprodigiosin (antibiotics, immunosuppressive, and anticancer)	S–NB	Luti and Mavituna (2011)
*Streptomyces* sp. Mg1	*Bacillus subtilis* 3610	Chalcomycin A (antibiotics)	S–NB	Barger et al. (2012)
*Streptomyces lividans*	*Bacillus subtilis* (bacillaene pks operon deletion)	Undecylprodigiosin (antibiotics, immunosuppressive, and anticancer)	S–NB	Vargas-Bautista et al. (2014)
*Streptomyces* sp.	*Bacillus mycoides*	Bacillamides A–C (algicidal)*N*-Acetyltryptamine (algicidal)*N*-Propanoyltryptamine (algicidal)	S–NB	Yu et al. (2015)
*Streptomyces coelicolor* M145	*Escherichia coli*	Undecylprodigiosin (antibiotics, immunosuppressive, and anticancer)	S–NB	Mavituna et al. (2016)
*Streptomyces coelicolor* M145	*Corallococcus coralloides* B035	Undecylprodigiosin (antibiotics, immunosuppressive, and anticancer)	S–NB	Schaberle et al. (2014)
*Streptomyces venezuelae* (methyltransferase from *S. avermitilis*)	Engineered *Escherichia coli*	*O*-Methylated phenylpropanoids (antibiotics)Multimethylated phenylpropanoids (antibiotics)	S–NB	Cui et al. (2019)
*Streptomyces griseorubiginosus* 43708	*Pseudomonas maltophilia* 1928	Biphenomycins A and C (antibiotics)	S–NB	Ezaki et al. (1992, 1993)
*Streptomyces tenjamariensis* SS-939 ATCC31603	12 unidentified bacteria	Istamycins A and B (antibiotics)	S–NB	Slattery et al. (2001)
*Streptomyces* sp. B033	*Brucella neotomae* ATCC 23459*Burkholderia vietnamiensis* ATCC BAA-248*Yersinia pestis* A1122*Xanthomonas axonopodis* ATCC 8718	Resistomycin (antibiotics)	S–NB	Carlson et al. (2015)
*Streptomyces cinnabarinus* PK209	*Alteromonas* sp. KNS-16	Lobocompactol (antifouling, antioxidant, and anticancer)	S–NB	Cho and Kim (2012)

S: *Streptomyces*; PRB: predatory bacteria; PAB: pathogenic bacteria; NB: non-actinomycetes bacteria.

Recently, transcriptome analysis on both *M. xanthus* and *S. coelicolor* during coculture revealed that iron competition between them, not physical contact, triggered actinorhodin production of *S. coelicolor* (Lee et al., 2020a). During coculture, *S. coelicolor* actively absorbed the extracellular iron, causing *M. xanthus* to face an iron-reduced environment. To respond to the iron-depletion condition, *M. xanthus* upregulated biosynthesis of siderophore, myxochelin, and myxochelin-mediated iron uptake systems, leading *M. xanthus* to dominate iron scavenging. Consequently, *S. coelicolor* experienced an iron-restricted condition and activated actinorhodin production along with upregulating branched amino acid catabolism, which implies the potential to produce precursors of actinorhodin. Based on these results, seven *Streptomyces* species (i.e., *S. subrutilus, S. kanamyceticus, S. coeruleorubidus, S. cinereoruber, S. roseosporus, S. rimosus*, and *S. venezuelae*) were cultured in iron-restricted conditions, resulting in upregulation of 21 smBGCs out of a total of 260 smBGCs in seven species’ genomes. Among secondary metabolites expected to be produced from upregulated smBGCs, several secondary metabolites, including actinorhodin, cosmomycin D, and chloramphenicol, possess putative iron-interacting sites, implying that these secondary metabolites might have both antibiotic and iron-chelating functions, which would be highly advantageous during iron competition with nearby microbes (Lee et al., 2020a).

#### Actinomycetes coculture with pathogenic bacteria

Human pathogenic bacteria such as *Staphylococcus aureus* have been tried to coculture with actinomycetes due to the advantage in real-time screening of induced secondary metabolites’ bioactivity against pathogenic bacteria (Table 2). For example, marine *Streptomyces* sp. PTY08712 was isolated from a complex tunicate community and cocultured with antibiotic-resistant human pathogens, including methicillin-sensitive *S. aureus*, methicillin-resistant *S. aureus* (MRSA), and *P. aeruginosa.* As a result, coculture extracts showed increased bioactivity against human pathogens, except *P. aeruginosa*, which results from enhanced production of three secondary metabolites: granatomycin D (antibacterial), granaticin (strong antibacterial), and dihydrogranaticin B (not known) (Sung et al., 2017). As with coculturing actinomycetes with stressors, coculturing with antibiotic-resistant pathogens could stimulate production of novel secondary metabolites effective to them. So far, why and how actinomycetes produce bioactive compounds against pathogenic bacteria have not been fully revealed, but from the example of *S. coelicolor* cultured with heat-killed pathogenic bacteria *S. aureus*, antibiotic producer *S. coelicolor* might recognize some proteins like receptors on the surface of pathogenic bacteria via physical cell-to-cell contact (Luti & Mavituna, 2011). Still, further revelation of mechanism is needed for elucidation of novel bioactive secondary metabolites from actinomycetes–pathogenic bacteria coculture.

Sometimes, actinomycetes require long-term microbial interaction to acquire the ability to produce antibiotics against nearby microbes. Continuous adaptive laboratory evolution (ALE) of *Streptomyces clavuligerus* implementing coculture with MRSA as a driving force resulted in *S. clavuligerus* acquiring the ability to constitutively produce a pyrrothine class of antibiotic, holomycin, which inhibits growth of MRSA. Competition between the two microbes led to genomic mutations of *S. clavuligerus*, including loss of megaplasmid and five single-nucleotide polymorphisms, which might affect the secondary metabolism (Charusanti et al., 2012). These results indicate that long-term coculture can activate silent smBGC by inducing genetic mutations, which keep silent under short-term coculture.

#### Actinomycetes coculture with other bacteria

Well-characterized model bacteria such as *B. subtilis* and *E. coli* have also been utilized as coculture partner with actinomycetes. For example, when *Streptomyces* sp. Mg1 was cocultured with competitor *B. subtilis*, chalcomycin A, which inhibits growth and even lyses *B. subtilis*, was produced by *Streptomyces* sp. Mg1 (Barger et al., 2012). Also, both live and heat-killed *B. subtilis* activated the undecylprodigiosin production of *S. coelicolor* and *S. lividans* (Luti & Mavituna, 2011; Straight et al., 2007; Vargas-Bautista et al., 2014). In addition, coculturing other *Bacillus* species such as *B. mycoides* and *B. cereus* with *Streptomyces* species activated production of bioactive secondary metabolites including algicides (e.g., bacillamide and tryptamines) (Yu et al., 2015) and antibiotics (e.g., dentigerumycin E), which are protective against *B. subtilis* (Shin et al., 2018). Meanwhile, in case of *E. coli*, when *S. coelicolor* was cocultured with live *E. coli* cell, undecylprodigiosin production of *S. coelicolor* was 3.5-fold increased, whereas actinorhodin production was 15-fold decreased. This secondary metabolism change was proven to be induced from the chemical compound in cell-free supernatant of *E. coli* (Mavituna et al., 2016).

Taken together, a broad range of bacteria has been utilized to trigger the production of numerous secondary metabolites from actinomycetes (Table 2). Predatory microbes and competitive participants, including human pathogens and model bacteria, have been cocultured with actinomycetes to induce production of defensive or inhibitory secondary metabolites, which have the potential for the development of antibiotics. Nevertheless, many of underlying principles of secondary metabolite production have not been clearly elucidated, which hinders further understanding of communications between actinomycetes and bacteria.

### Actinomycetes Cocultured With Fungi

#### Actinomycetes as a producer

Fungal species have been revealed to possess about 50 cryptic smBGCs per genome like actinomycetes (Nierman et al., 2005; Pel et al., 2007; Wortman et al., 2009) and many fungi coexist with actinomycetes in various ecological habitats, implying inter-kingdom interactions between them are commonly present (Frey-Klett et al., 2011; Hibbing et al., 2010; Kroiss et al., 2010). Indeed, interaction between actinomycetes and fungi activated the secondary metabolism of actinomycetes (Table 3). For example, when *S. lividans* was cocultured with *Verticillium dahlia*, the production of the antibiotic undecylprodigiosin was upregulated. Undecylprodigiosin strongly reduced the microsclerotia formation of *V. dahlia*, possibly by interfering with the signal transduction pathway (Meschke et al., 2012). Another example is that coculturing *Streptomyces leeuwenhoekii* C58 with *Aspergillus fumigatus* MR2012 in various culture media induced the production of nocardamine, pentalenic acid, and chaxapeptin by *S. leeuwenhoekii* C58, but none of these metabolites were proved to have antifungal bioactivity (Wakefield et al., 2017).

**Table 3.** Actinomycetes and Fungi Coculture

Producer	Inducer	Induced compounds and bioactivity	Category (producer–inducer)	References
*Streptomyces lividans*	*Verticillium dahliae*	Undecylprodigiosin (antifungal)	S–F	Meschke et al. (2012)
*Streptomyces leeuwenhoekii* C58	*Aspergillus fumigatus* MR2012	Chaxapeptin	S–F	Wakefield et al. (2017)
*Streptomyces rochei* MB037	*Rhinocladiella similis* 35	Borrelidin J (antibiotics)Borrelidins K and F 7-Methoxy-2,3-dimethylchromone-4-one	S–F	Yu et al. (2019)
*Aspergillus nidulans*	*Streptomyces rapamycinicus*	Orsellinic acidLecanoric acid (inhibit ATP synthesis)Cathepsin K inhibitors F-9775A and F-9775B (antiosteoporosis)	F–S	Fischer et al. (2018), Nutzmann et al. (2011), and Schroeckh et al. (2009)
*Aspergillus fumigatus*	*Streptomyces rapamycinicus*	Fumicyclines A and B (antibiotics)	F–S	Konig et al. (2013)
*Aspergillus fumigatus*	*Streptomyces rapamycinicusStreptomyces iranensisStreptomyces coelicolorStreptomyces lividans*	Fumigermin (antibiotics)	F–S	Stroe et al. (2020)
*Aspergillus fumigatus*	*Streptomyces peucetius* ATCC 29050	Fumiformamide*N,N*′-[(1*Z*,3*Z*)-1,4-Bis(4-methoxyphenyl)buta-1,3-diene-2,3-diyl]diformamide (cytotoxicity)*N*-Formyl derivatives (cytotoxicity)BU-4704Xanthocillin X monoetherXanthocillin X dietherXanthocillin dimethyl etherXanthoascin	F–S	Zuck et al. (2011)
*Aspergillus fumigatus* MBC-F1-10	*Streptomyces bullii*	ErgosterolBrevianamide F (cytotoxicity)Spirotryprostatin A (antibiotics)6-Methoxyspirotryprostatin B (leishmanicidal and cytotoxicity)Fumitremorgin C (antitrypanosomal and leishmanicidal)12,13-Dihydroxyfumitremorgin C (antitrypanosomal and leishmanicidal)Fumitremorgin B (antitrypanosomal and leishmanicidal)Verruculogen (antitrypanosomal and leishmanicidal)11-*O*-Methylpseurotin A (cytotoxicity)11-*O*-Methylpseurotin A2 (antitrypanosomal and leishmanicidal)Emestrins A and B (induced by quorum-sensing molecule)	F–S	Rateb et al. (2013)
*Aspergillus fumigatus* MR2012	*Streptomyces leeuwenhoekii* C34	Luteoride DPseurotin GTerezine D11-*O*-Methylpseurotin A Chaxapeptin	F–S	Wakefield et al. (2017)
*Aspergillus niger*	*Streptomyces coelicolor*	*cyclo*-(Phe–Phe)*cyclo*-(Phe–Tyr)Phenylacetic acid2-HydroxyphenylaceticFuran-2-carboxylic acid	F–S	Wu et al. (2015)
*Aspergillus flavipes*	*Streptomyces* sp.	RosellichalasinAspochalasins E, P, H, and M19,20-Dihydro-aspochalasin D	F–S	Yu et al. (2016)
*Aspergillus austroafricanus*	*Streptomyces lividans*	AustramideViolaceols I and II (antibiotics)Diorcinol (antibiotics)	F–S	Ebrahim et al. (2016)
*Aspergillus* sp.	*Streptomyces* sp.	Heronapyrrole B from *Streptomyces* (antifungal)Debromomarinone*cyclo*-(l-Phe-*trans*-4-hydroxy-l-Pro) from *Aspergillus* (antibiotics)	F–S	Khalil et al. (2019)
*Fusarium tricinctum*	*Streptomyces lividans*	Fusatricinones A–DDihydrolateropyroneLateropyrone (antibiotics)Zearalenone(−)-CitreoisocoumarinMacrocarpon C7-Hydroxy-2-(2-hydroxypropyl)-5-methylchromoneDepsipeptide enniatins A1, B, and B1 (antibiotics)Lipopeptide fusaristatin A	F–S	Moussa et al. (2019)
*Bionectria* sp.	*Streptomyces lividans* TK24	Bionectriamines A and BTris(2,4-di-*tert*-butylphenyl) phosphate6,8-Dihydroxyisocoumarin-3-carboxylic acid	F–S	Kamdem et al. (2018)
*Penicillium* sp. WC-29-5	*Streptomyces fradiae* 007	DeoxyfuniconeAlternariolVermistatin(9*R*,14*S*)-Epoxy-11-deoxyfunicone (cytotoxicity)(9*S*,14*R*)-Epoxy-11-deoxyfunicone (cytotoxicity)	F–S	Wang et al. (2014)
*Heterobasidion abietinum* 331	*Streptomyces* AcH 505	5-Formylsalicylic acid (virulence factor and siderophore)	F–S	Keilhofer et al. (2018)
*Emericella* sp. CNL-878	*Salinispora arenicola* sp. CNH-665	Emericellamides A and B (anticancer)	F–S	Oh et al. (2007)

S: *Streptomyces*; F: fungi.

#### Fungi as a producer

Unlike the above examples, in most cases of actinomycetes–fungi interactions, fungi, especially *Aspergillus* species, act as producers while actinomycetes induce the secondary metabolism of fungi (Table 3). Coculturing *Aspergillus nidulans* with a collection of 58 soil-dwelling actinomycetes is a representative example of activating silent fungal smBGCs by coculture with actinomycetes. As a result, four secondary metabolites (orsellinic acid [OA], lecanoric acid [LA], F-9775A, and F-9775B) were produced from *A. nidulans* only when cocultured with *Streptomyces hygroscopicus* (renamed as *Streptomyces rapamycinicus*). Interestingly, further analysis revealed that physical interaction between *A. nidulans* and *S. rapamycinicus* is required for inducing the secondary metabolism of *A. nidulans* (Schroeckh et al., 2009). It was discovered that physical contact between the two organisms triggered histone acetylation of the OA-encoding *ors* gene of *A. nidulans* by histone acetyltransferase Saga/Ada complex, ultimately inducing OA and LA production (Nutzmann et al., 2011). The latest study elucidated that transcriptional factor BasR acts as a central “node” for linking external signals from physical interaction with actinomycetes and secondary metabolic regulation, including OA production (Fischer et al., 2018). In addition, the fungal species *A. fumigatus* was cocultured with the inducer *S. rapamycinicus*, resulting in the production of fungal secondary metabolites fumicyclines A and B and fumigermin (Konig et al., 2013; Stroe et al., 2020). In the case of fumicyclines A and B, the same principle of histone modifications was working when *S. rapamycinicus* was cocultured with *A. fumigatus*, whereas in the case of fumigermin, it was not clarified whether elicitation was via histone modification or not (Konig et al., 2013; Stroe et al., 2020). As for the bioactivity of induced compounds, fumicyclines A and B showed antibacterial effect to *S. rapamycinicus* and fumigermin inhibited germination of *S. rapamycinicus*, indicating that compounds induced during coculture with *S. rapamycinicus* are considered as fungal defensive systems.

We categorized actinomycetes–fungi coculture into two sections: (i) actinomycetes as a producer and (ii) fungi as a producer. Despite this, chemical and physical interactions between the two kingdoms often cause complex metabolic shifts of both organisms to produce various secondary metabolites as a defensive response (Table 3). Considering that most of the aforementioned cases have been focused on analyzing a few induced secondary metabolites, it is expected that there may have been more diverse alterations in secondary metabolite production than reported. For example, *Aspergillus* sp. CMB-StM0423 produces a bacteriostatic compound, diketopiperazine, when cocultured with *Streptomyces* sp. CMB-StM0423 (Khalil et al., 2019). Actually, diketopiperazines are common secondary metabolites and are known to be overproduced by *Aspergillus* when cocultured with *Streptomyces* (Wakefield et al., 2017; Wu et al., 2015). Transcriptome analysis revealed that diketopiperazine stimulated *Streptomyces* to repress nitric oxide (NO) dioxygenase, which reduced the level of NO gas in the cell, resulting in a high intracellular concentration of NO gas. As a result, a high concentration of NO-activated novel smBGCs and antifungal compound, heronapyrrole B, was produced by *Streptomyces* (Khalil et al., 2019). In addition, when marine-derived *Streptomyces rochei* MB037 was cultured with the fungi *Rhinocladiella similis*, two novel antibacterial borrelidins, J and K, were produced by *S. rochei* and one antibacterial chromone was produced by *R. similis* (Yu et al., 2019).

Overall, actinomycetes act as both inducer and producer when cocultured with various fungal species (Table 3). In some instances, actinomycetes trigger epigenetic modification of fungi, resulting in complex secondary metabolism changes, and sometimes fungi produce certain secondary metabolites, which alter the secondary metabolism of actinomycetes. The interaction between fungi and actinomycetes is mainly attack and defense, so if coculturing pathogenic actinomycetes or pathogenic fungi, it seems likely novel secondary metabolites that can kill each other will be discovered.

## Conclusion

To date, numerous bioactive secondary metabolites have been elicited through coculture of actinomycetes with various bacteria or fungi. Coculture provides complex stimuli, which dramatically affect secondary metabolism of actinomycetes, and allows the real-time bioactivity screening of newly induced secondary metabolites; thus, it is highly advantageous to the discovery of novel bioactive secondary metabolites with triggering mechanisms. However, the coculture method is often irreproducible and inappropriate for large-scale culture to produce target secondary metabolites abundantly. Yet, the secondary metabolite induction stimuli elucidated from coculture study can be exploited in industrial applications for secondary metabolite production by single culture. Thus, a precise and comprehensive understanding of the underlying coculture mechanism is a top priority (Lee et al., 2020a).

After examining the previous reports in an effort to discover the underlying principles of coculture, induction mechanisms can be categorized into three scenarios (Fig. 1): (i) physical interactions, (ii) chemical communications (e.g., nutrient competition and quorum sensing), and (iii) genomic alteration (e.g., horizontal gene transfer and genomic mutation by ALE). However, still only a few in-depth studies about the genetic regulatory network linked with those inducing signals. For example, physical cell-to-cell interactions between fungi and *Streptomyces* triggered chromosome acetylation of fungi, which implies not just physical interaction itself but also a further underlying mechanism to bring out the secondary metabolism changes (Nutzmann et al., 2011). In recent years, various tools have been developed and applied for elucidating these inducing mechanisms during coculture. In particular, transcriptomic analysis enables the examination of the genetic responses of each coculture participant. Functional analysis of differently expressed genes during coculture allows tracing the triggering factors and responses of producer and inducer. In addition, comparative proteomic and metabolic analysis between axenic culture and coculture enables the clarification of the dynamics of proteins and molecules related to secondary metabolism. Multi-omics technology-based mechanical studies on the coculture will improve our understanding of the secondary metabolic regulation of actinomycetes.

Moreover, previous cocultures of actinomycetes were limited in range of culture partner, which may have restricted the range of secondary metabolism involved; therefore, coculture with more diverse partners, such as amoeba or phages, is needed (Klapper et al., 2016; Kronheim et al., 2018). For instance, coculturing actinomycetes with double-stranded DNA phages unveiled a secondary metabolism of *Streptomyces* involved in defense against phage infection (Kronheim et al., 2018). Accumulation of diverse microbial coculture studies will help us to understand the relationship between coculture conditions (e.g., coculture partner, culture media, and culture type) and type of induced secondary metabolites. Indeed, recent comprehensive analysis demonstrating the induction of 259 compounds via coculture revealed that production of “linear polyketides, oxylipins, and fatty acids” and “cyclic peptides, diketopiperazines, and related compounds” seems to occur mostly during liquid fermentation compared to solid coculture, independent of the type of coculture participants (Arora et al., 2020). As pointed out in the study, lack of information provided by previous coculture studies is the main hurdle to comprehensive understanding; thus, general guidelines are needed for the coculture studies to provide accurate and sufficient information.

In conclusion, numerous coculture studies have successfully discovered novel secondary metabolites from actinomycetes to date, but even so, the precise mechanisms of interaction are rarely understood. Broader and deeper identification of the inducing mechanisms during coculture is required to understand complex secondary metabolic regulation and to set directions to genetic engineering-based strategies for inducing or increasing production of target secondary metabolites.

## References

[bib1] Abdelmohsen U. R., Grkovic T., Balasubramanian S., Kamel M. S., Quinn R. J., Hentschel U. (2015). Elicitation of secondary metabolism in actinomycetes. Biotechnology Advances, 33, 798–811. 10.1016/j.biotechadv.2015.06.003 26087412

[bib2] Adnani N., Chevrette M. G., Adibhatla S. N., Zhang F., Yu Q., Braun D. R., Nelson J., Simpkins S. W., McDonald B. R., Myers C. L., Piotrowski J. S., Thompson C. J., Currie C. R., Li L., Rajski S. R., Bugni T. S. (2017). Coculture of marine invertebrate-associated bacteria and interdisciplinary technologies enable biosynthesis and discovery of a new antibiotic, keyicin. ACS Chemical Biology, 12, 3093–3102. 10.1021/acschembio.7b00688. 29121465 PMC5973552

[bib3] Adnani N., Vazquez-Rivera E., Adibhatla S. N., Ellis G. A., Braun D. R., Bugni T. S. (2015). Investigation of interspecies interactions within marine *Micromonosporaceae* using an improved co-culture approach. Marine Drugs, 13, 6082–6098. 10.3390/md13106082 26404321 PMC4626680

[bib4] Amano S., Morota T., Kano Y. K., Narita H., Hashidzume T., Yamamoto S., Mizutani K., Sakuda S., Furihata K., Takano-Shiratori H., Takano H., Beppu T., Ueda K. (2010). Promomycin, a polyether promoting antibiotic production in *Streptomyces* spp. Journal of Antibiotics, 63, 486–491. 10.1038/ja.2010.68 20571515

[bib5] Aminov R. I. (2010). A brief history of the antibiotic era: Lessons learned and challenges for the future. Frontiers in Microbiology, 1, 134. 10.3389/fmicb.2010.00134 21687759 PMC3109405

[bib6] Arora D., Gupta P., Jaglan S., Roullier C., Grovel O., Bertrand S. (2020). Expanding the chemical diversity through microorganisms co-culture: Current status and outlook. Biotechnology Advances, 40, 107521. 10.1016/j.biotechadv.2020.107521 31953204

[bib7] Baltz R. H. (2008). Renaissance in antibacterial discovery from actinomycetes. Current Opinion in Pharmacology, 8, 557–563. 10.1016/j.coph.2008.04.008 18524678

[bib8] Barger S. R., Hoefler B. C., Cubillos-Ruiz A., Russell W. K., Russell D. H., Straight P. D. (2012). Imaging secondary metabolism of *Streptomyces* sp. Mg1 during cellular lysis and colony degradation of competing *Bacillus subtilis*. Antonie Van Leeuwenhoek, 102, 435–445. 10.1007/s10482-012-9769-0 22777252

[bib9] Belknap K. C., Park C. J., Barth B. M., Andam C. P. (2020). Genome mining of biosynthetic and chemotherapeutic gene clusters in *Streptomyces* bacteria. Scientific Reports, 10, 2003. 10.1038/s41598-020-58904-9 32029878 PMC7005152

[bib10] Bhatnagar R. K., Doull J. L., Vining L. C. (1988). Role of the carbon source in regulating chloramphenicol production by *Streptomyces venezuelae*: Studies in batch and continuous cultures. Canadian Journal of Microbiology, 34, 1217–1223. 10.1139/m88-214 3208198

[bib11] Bibb M. J. (2005). Regulation of secondary metabolism in *Streptomycetes*. Current Opinion in Microbiology, 8, 208–215. 10.1016/j.mib.2005.02.016 15802254

[bib12] Bode H. B., Bethe B., Hofs R., Zeeck A. (2002). Big effects from small changes: Possible ways to explore nature's chemical diversity. ChemBioChem, 3, 619–627. 10.1002/1439-7633(20020703)3:7<619::Aid-Cbic619>3.0.Co;2-9 12324995

[bib13] Boucher H. W., Talbot G. H., Bradley J. S., Edwards J. E., Gilbert D., Rice L. B., Scheld M., Spellberg B., Bartlett J. (2009). Bad bugs, no drugs: No ESKAPE! An update from the Infectious Diseases Society of America. Clinical Infectious Diseases, 48, 1–12. 10.1086/595011 19035777

[bib14] Carlson S., Tanouye U., Omarsdottir S., Murphy B. T. (2015). Phylum-specific regulation of resistomycin production in a *Streptomyces* sp. via microbial coculture. Journal of Natural Products, 78, 381–387. 10.1021/np500767u 25537064

[bib15] Challis G. L., Hopwood D. A. (2003). Synergy and contingency as driving forces for the evolution of multiple secondary metabolite production by *Streptomyces* species. Proceedings of the National Academy of Sciences of the USA, 100, 14555–14561. 10.1073/pnas.1934677100 12970466 PMC304118

[bib16] Charusanti P., Fong N. L., Nagarajan H., Pereira A. R., Li H. J., Abate E. A., Su Y., Gerwick W. H., Palsson B. O. (2012). Exploiting adaptive laboratory evolution of *Streptomyces clavuligerus* for antibiotic discovery and overproduction. PLoS One, 7, e33727. 10.1371/journal.pone.0033727 22470465 PMC3312335

[bib17] Chen G., Wang G. Y., Li X., Waters B., Davies J. (2000). Enhanced production of microbial metabolites in the presence of dimethyl sulfoxide. Journal of Antibiotics, 53, 1145–1153. 10.7164/antibiotics.53.1145 11132960

[bib18] Cho J. Y., Kim M. S. (2012). Induction of antifouling diterpene production by *Streptomyces cinnabarinus* PK209 in co-culture with marine-derived *Alteromonas* sp. KNS-16. Bioscience, Biotechnology, and Biochemistry, 76, 1849–1854. 10.1271/bbb.120221 23047094

[bib19] Christova K., Sholeva Z., Chipeva V. (1995). Application of molecular biological methods in taxonomy of genus *Streptomyces*. Journal of Culture Collections, 1, 3–10.

[bib20] Cihak M., Kamenik Z., Smidova K., Bergman N., Benada O., Kofronova O., Petrickova K., Bobek J. (2017). Secondary metabolites produced during the germination of *Streptomyces coelicolor*. Frontiers in Microbiology, 8, 2495. 10.3389/fmicb.2017.02495 29326665 PMC5733532

[bib21] Cobb R. E., Wang Y., Zhao H. (2015). High-efficiency multiplex genome editing of *Streptomyces* species using an engineered CRISPR/Cas system. ACS Synthetic Biology, 4, 723–728. 10.1021/sb500351f 25458909 PMC4459934

[bib22] Craney A., Ahmed S., Nodwell J. (2013). Towards a new science of secondary metabolism. Journal of Antibiotics, 66, 387–400. 10.1038/ja.2013.25 23612726

[bib23] Cui H., Song M. C., Ban Y. H., Jun S. Y., Kwon A. S., Lee J. Y., Yoon Y. J. (2019). High-yield production of multiple *O*-methylated phenylpropanoids by the engineered *Escherichia coli*–*Streptomyces* cocultivation system. Microbial Cell Factories, 18, 67. 10.1186/s12934-019-1118-9 30971246 PMC6456975

[bib24] Dashti Y., Grkovic T., Abdelmohsen U. R., Hentschel U., Quinn R. J. (2014). Production of induced secondary metabolites by a co-culture of sponge-associated actinomycetes, *Actinokineospora* sp. EG49 and *Nocardiopsis* sp. RV163. Marine Drugs, 12, 3046–3059. 10.3390/md12053046 24857962 PMC4052330

[bib25] Derewacz D. K., Covington B. C., McLean J. A., Bachmann B. O. (2015). Mapping microbial response metabolomes for induced natural product discovery. ACS Chemical Biology, 10, 1998–2006. 10.1021/acschembio.5b00001 26039241 PMC4987304

[bib26] Doroghazi J. R., Albright J. C., Goering A. W., Ju K. S., Haines R. R., Tchalukov K. A., Labeda D. P., Kelleher N. L., Metcalf W. W. (2014). A roadmap for natural product discovery based on large-scale genomics and metabolomics. Nature Chemical Biology, 10, 963–968. 10.1038/nchembio.1659. 25262415 PMC4201863

[bib27] Ebrahim W., El-Neketi M., Lewald L. I., Orfali R. S., Lin W. H., Rehberg N., Kalscheuer R., Daletos G., Proksch P. (2016). Metabolites from the fungal endophyte *Aspergillus austroafricanus* in axenic culture and in fungal-bacterial mixed cultures. Journal of Natural Products, 79, 914–922. 10.1021/acs.jnatprod.5b00975 27070198

[bib28] Ezaki M., Iwami M., Yamashita M., Komori T., Umehara K., Imanaka H. (1992). Biphenomycin A production by a mixed culture. Applied and Environmental Microbiology, 58, 3879–3882. 1476432 10.1128/aem.58.12.3879-3882.1992 PMC183198

[bib29] Ezaki M., Shigematsu N., Yamashita M., Komori T., Umehara K., Imanaka H. (1993). Biphenomycin C, a precursor of biphenomycin A in mixed culture. Journal of Antibiotics, 46, 135–140. 10.7164/antibiotics.46.135 8436546

[bib30] Fischer J., Muller S. Y., Netzker T., Jager N., Gacek-Matthews A., Scherlach K., Stroe M. C., Garcia-Altares M., Pezzini F., Schoeler H., Reichelt M., Gershenzon J., Krespach M. K., Shelest E., Schroeckh V., Valiante V., Heinzel T., Hertweck C., Strauss J., Brakhage A. A. (2018). Chromatin mapping identifies BasR, a key regulator of bacteria-triggered production of fungal secondary metabolites. eLife, 7, e40969. 10.7554/eLife.40969 30311911 PMC6234034

[bib31] Frey-Klett P., Burlinson P., Deveau A., Barret M., Tarkka M., Sarniguet A. (2011). Bacterial–fungal interactions: Hyphens between agricultural, clinical, environmental, and food microbiologists. Microbiology and Molecular Biology Reviews, 75, 583–609. 10.1128/Mmbr.00020-11 22126995 PMC3232736

[bib32] Gao C., Hindra, Mulder D., Yin C., Elliot M. A. (2012). Crp is a global regulator of antibiotic production in *Streptomyces*. mBio, 3, e00407–12. 10.1128/mBio.00407-12 23232715 PMC3520106

[bib33] Grafe U., Reinhardt G., Schade W., Eritt I., Fleck W. F., Radics L. (1983). Interspecific inducers of cytodifferentiation and anthracycline biosynthesis from *Streptomyces bikinensis* and *Streptomyces cyaneofuscatus*. Biotechnology Letters, 5, 591–596. 10.1007/Bf00130838

[bib34] Han J., Gao Q. X., Zhang Y. G., Li L., Mohamad O. A. A., Rao M. P. N., Xiao M., Hozzein W. N., Alkhalifah D. H. M., Tao Y., Li W. J. (2018). Transcriptomic and ectoine analysis of halotolerant *Nocardiopsis gilva* YIM 90087(T) under salt stress. Frontiers in Microbiology, 9, 618. 10.3389/fmicb.2018.00618 29651284 PMC5884947

[bib35] Hara O., Beppu T. (1982). Induction of streptomycin-inactivating enzyme by A-factor in *Streptomyces griseus*. Journal of Antibiotics, 35, 1208–1215. 10.7164/antibiotics.35.1208 6292150

[bib36] Harvey A. L. (2008). Natural products in drug discovery. Drug Discovery Today, 13, 894–901. 10.1016/j.drudis.2008.07.004 18691670

[bib37] Hibbing M. E., Fuqua C., Parsek M. R., Peterson S. B. (2010). Bacterial competition: Surviving and thriving in the microbial jungle. Nature Reviews Microbiology, 8, 15–25. 10.1038/nrmicro2259 19946288 PMC2879262

[bib38] Horinouchi S., Beppu T. (1992). Autoregulatory factors and communication in actinomycetes. Annual Review of Microbiology, 46, 377–398. 10.1146/annurev.mi.46.100192.002113 1444261

[bib39] Hosaka T., Ohnishi-Kameyama M., Muramatsu H., Murakami K., Tsurumi Y., Kodani S., Yoshida M., Fujie A., Ochi K. (2009). Antibacterial discovery in actinomycetes strains with mutations in RNA polymerase or ribosomal protein S12. Nature Biotechnology, 27, 462–464. 10.1038/nbt.1538 19396160

[bib41] Hoshino S., Okada M., Awakawa T., Asamizu S., Onaka H., Abe I. (2017). Mycolic acid containing bacterium stimulates tandem cyclization of polyene macrolactam in a lake sediment derived rare actinomycete. Organic Letters, 19, 4992–4995. 10.1021/acs.orglett.7b02508 28880091

[bib42] Hoshino S., Okada M., Wakimoto T., Zhang H., Hayashi F., Onaka H., Abe I. (2015a). Niizalactams A–C, multicyclic macrolactams isolated from combined culture of *Streptomyces* with mycolic acid-containing bacterium. Journal of Natural Products, 78, 3011–3017. 10.1021/acs.jnatprod.5b00804 26624939

[bib43] Hoshino S., Ozeki M., Awakawa T., Morita H., Onaka H., Abe I. (2018a). Catenulobactins A and B, heterocyclic peptides from culturing *Catenuloplanes* sp. with a mycolic acid-containing bacterium. Journal of Natural Products, 81, 2106–2110. 10.1021/acs.jnatprod.8b00261 30130105

[bib44] Hoshino S., Ozeki M., Wong C. P., Zhang H., Hayashi F., Awakawa T., Morita H., Onaka H., Abe I. (2018b). Mirilactams C–E, novel polycyclic macrolactams isolated from combined-culture of *Actinosynnema mirum* NBRC 14064 and mycolic acid-containing bacterium. Chemical and Pharmaceutical Bulletin, 66, 660–667. 10.1248/cpb.c18-00143 29863068

[bib40] Hoshino S., Wakimoto T., Onaka H., Abe I. (2015b). Chojalactones A–C, cytotoxic butanolides isolated from *Streptomyces* sp. cultivated with mycolic acid containing bacterium. Organic Letters, 17, 1501–1504. 10.1021/acs.orglett.5b00385 25742189

[bib45] Hoshino S., Wong C. P., Ozeki M., Zhang H., Hayashi F., Awakawa T., Asamizu S., Onaka H., Abe I. (2018c). Umezawamides, new bioactive polycyclic tetramate macrolactams isolated from a combined-culture of *Umezawaea* sp. and mycolic acid-containing bacterium. Journal of Antibiotics, 71, 653–657. 10.1038/s41429-018-0040-4 29540776

[bib46] Hoshino S., Zhang L., Awakawa T., Wakimoto T., Onaka H., Abe I. (2015c). Arcyriaflavin E, a new cytotoxic indolocarbazole alkaloid isolated by combined-culture of mycolic acid-containing bacteria and *Streptomyces cinnamoneus* NBRC 13823. Journal of Antibiotics, 68, 342–344. 10.1038/ja.2014.147 25335694

[bib47] Huang H., Zheng G., Jiang W., Hu H., Lu Y. (2015). One-step high-efficiency CRISPR/Cas9-mediated genome editing in *Streptomyces*. Acta Biochimica et Biophysica Sinica, 47, 231–243. 10.1093/abbs/gmv007 25739462

[bib48] Jose P. A., Jebakumar S. R. (2012). Phylogenetic diversity of actinomycetes cultured from coastal multipond solar saltern in Tuticorin, India. Aquatic Biosystems, 8, 23. 10.1186/2046-9063-8-23 22950748 PMC3496644

[bib49] Kamdem R. S. T., Wang H., Wafo P., Ebrahim W., Ozkaya F. C., Makhloufi G., Janiak C., Sureechatchaiyan P., Kassack M. U., Lin W. H., Liu Z., Proksch P. (2018). Induction of new metabolites from the endophytic fungus *Bionectria* sp. through bacterial co-culture. Fitoterapia, 124, 132–136. 10.1016/j.fitote.2017.10.021 29106994

[bib50] Kawai K., Wang G., Okamoto S., Ochi K. (2007). The rare earth, scandium, causes antibiotic overproduction in *Streptomyces* spp. FEMS Microbiology Letters, 274, 311–315. 10.1111/j.1574-6968.2007.00846.x 17645525

[bib51] Keilhofer N., Nachtigall J., Kulik A., Ecke M., Hampp R., Sussmuth R. D., Fiedler H. P., Schrey S. D. (2018). *Streptomyces* AcH 505 triggers production of a salicylic acid analogue in the fungal pathogen *Heterobasidion abietinum* that enhances infection of Norway spruce seedlings. Antonie Van Leeuwenhoek, 111, 691–704. 10.1007/s10482-018-1017-9 29350358

[bib52] Khalil Z. G., Cruz-Morales P., Licona-Cassani C., Marcellin E., Capon R. J. (2019). Inter-kingdom beach warfare: Microbial chemical communication activates natural chemical defences. ISME Journal, 13, 147–158. 10.1038/s41396-018-0265-z 30116041 PMC6299108

[bib53] Khokhlov A. S., Anisova L. N., Tovarova I. I., Kleiner E. M., Kovalenko I. V., Krasilnikova O. I., Kornitskaya E. Y., Pliner S. A. (1973). Effect of A-factor on the growth of asporogenous mutants of *Streptomyces griseus*, not producing this factor. Zeitschrift fur Allgemeine Mikrobiologie, 13, 647–655. 10.1002/jobm.3630130803 4131140

[bib54] Kim E. S., Hong H. J., Choi C. Y., Cohen S. N. (2001). Modulation of actinorhodin biosynthesis in *Streptomyces lividans* by glucose repression of *afsR2* gene transcription. Journal of Bacteriology, 183, 2198–2203. 10.1128/JB.183.7.2198-2203.2001 11244057 PMC95124

[bib55] Klapper M., Gotze S., Barnett R., Willing K., Stallforth P. (2016). Bacterial alkaloids prevent amoebal predation. Angewandte Chemie, International Edition, 55, 8944–8947. 10.1002/anie.201603312 27294402

[bib56] Koehn F. E., Carter G. T. (2005). The evolving role of natural products in drug discovery. Nature Reviews Drug Discovery, 4, 206–220. 10.1038/nrd1657 15729362

[bib57] Konig C. C., Scherlach K., Schroeckh V., Horn F., Nietzsche S., Brakhage A. A., Hertweck C. (2013). Bacterium induces cryptic meroterpenoid pathway in the pathogenic fungus *Aspergillus fumigatus*. ChemBioChem, 14, 938–942. 10.1002/cbic.201300070 23649940

[bib58] Kroiss J., Kaltenpoth M., Schneider B., Schwinger M. G., Hertweck C., Maddula R. K., Strohm E., Svatos A. (2010). Symbiotic *Streptomycetes* provide antibiotic combination prophylaxis for wasp offspring. Nature Chemical Biology, 6, 261–263. 10.1038/Nchembio.331 20190763

[bib59] Kronheim S., Daniel-Ivad M., Duan Z., Hwang S., Wong A. I., Mantel I., Nodwell J. R., Maxwell K. L. (2018). A chemical defence against phage infection. Nature, 564, 283–286. 10.1038/s41586-018-0767-x 30518855

[bib60] Kurosawa K., Ghiviriga I., Sambandan T. G., Lessard P. A., Barbara J. E., Rha C., Sinskey A. J. (2008). Rhodostreptomycins, antibiotics biosynthesized following horizontal gene transfer from *Streptomyces padanus* to *Rhodococcus fascians*. Journal of the American Chemical Society, 130, 1126–1127. 10.1021/ja077821p 18179219

[bib61] Laureti L., Song L., Huang S., Corre C., Leblond P., Challis G. L., Aigle B. (2011). Identification of a bioactive 51-membered macrolide complex by activation of a silent polyketide synthase in *Streptomyces ambofaciens*. Proceeding of the National Academy of Sciences of the USA, 108, 6258–6263. 10.1073/pnas.1019077108 PMC3076887 21444795

[bib62] Lee N., Kim W., Chung J., Lee Y., Cho S., Jang K. S., Kim S. C., Palsson B., Cho B. K. (2020a). Iron competition triggers antibiotic biosynthesis in *Streptomyces coelicolor* during coculture with *Myxococcus xanthus*. ISME Journal, 14, 1111–1124. 10.1038/s41396-020-0594-6 31992858 PMC7174319

[bib63] Lee N., Kim W., Hwang S., Lee Y., Cho S., Palsson B., Cho B. K. (2020b). Thirty complete *Streptomyces* genome sequences for mining novel secondary metabolite biosynthetic gene clusters. Scientific Data, 7, 55. 10.1038/s41597-020-0395-9 32054853 PMC7018776

[bib64] Luo Y., Huang H., Liang J., Wang M., Lu L., Shao Z., Cobb R. E., Zhao H. (2013). Activation and characterization of a cryptic polycyclic tetramate macrolactam biosynthetic gene cluster. Nature Communications, 4, 2894. 10.1038/ncomms3894 PMC3969335 24305602

[bib65] Luti K. J., Mavituna F. (2011). Elicitation of *Streptomyces coelicolor* with dead cells of *Bacillus subtilis* and *Staphylococcus aureus* in a bioreactor increases production of undecylprodigiosin. Applied Microbiology and Biotechnology, 90, 461–466. 10.1007/s00253-010-3032-2 21222119

[bib66] Mavituna F., Luti K. J. K., Gu L. X. (2016). In search of the *E. coli* compounds that change the antibiotic production pattern of *Streptomyces coelicolor* during inter-species interaction. Enzyme and Microbial Technology, 90, 45–52. 10.1016/j.enzmictec.2016.03.009 27241291

[bib67] Meschke H., Walter S., Schrempf H. (2012). Characterization and localization of prodiginines from *Streptomyces lividans* suppressing *Verticillium dahliae* in the absence or presence of *Arabidopsis thaliana*. Environmental Microbiology, 14, 940–952. 10.1111/j.1462-2920.2011.02665.x 22151498

[bib68] Moussa M., Ebrahim W., Bonus M., Gohlke H., Mandi A., Kurtan T., Hartmann R., Kalscheuer R., Lin W. H., Liu Z., Proksch P. (2019). Co-culture of the fungus *Fusarium tricinctum* with *Streptomyces lividans* induces production of cryptic naphthoquinone dimers. RSC Advances, 9, 1491–1500. 10.1039/c8ra09067j 35518011 PMC9060880

[bib69] Nett M., Ikeda H., Moore B. S. (2009). Genomic basis for natural product biosynthetic diversity in the actinomycetes. Natural Product Reports, 26, 1362–1384. 10.1039/b817069j 19844637 PMC3063060

[bib70] Nierman W. C., Pain A., Anderson M. J., Wortman J. R., Kim H. S., Arroyo J., Berriman M., Abe K., Archer D. B., Bermejo C., Bennett J., Bowyer P., Chen D., Collins M., Coulsen R., Davies R., Dyer P. S., Farman M., Fedorova N., Denning D. W. (2005). Genomic sequence of the pathogenic and allergenic filamentous fungus *Aspergillus fumigatus*. Nature, 438, 1151–1156. 10.1038/nature04332 16372009

[bib71] Niu G., Chater K. F., Tian Y., Zhang J., Tan H. (2016). Specialised metabolites regulating antibiotic biosynthesis in *Streptomyces* spp. FEMS Microbiology Reviews, 40, 554–573. 10.1093/femsre/fuw012 27288284

[bib72] Nutzmann H. W., Reyes-Dominguez Y., Scherlach K., Schroeckh V., Horn F., Gacek A., Schumann J., Hertweck C., Strauss J., Brakhage A. A. (2011). Bacteria-induced natural product formation in the fungus *Aspergillus nidulans* requires Saga/Ada-mediated histone acetylation. Proceedings of the National Academy of Sciences of the USA, 108, 14282–14287. 10.1073/pnas.1103523108 21825172 PMC3161617

[bib73] Oh D. C., Kauffman C. A., Jensen P. R., Fenical W. (2007). Induced production of emericellamides A and B from the marine-derived fungus *Emericella* sp. in competing co-culture. Journal of Natural Products, 70, 515–520. 10.1021/np060381f 17323993

[bib74] Ohnishi Y., Ishikawa J., Hara H., Suzuki H., Ikenoya M., Ikeda H., Yamashita A., Hattori M., Horinouchi S. (2008). Genome sequence of the streptomycin-producing microorganism *Streptomyces griseus* IFO 13350. Journal of Bacteriology, 190, 4050–4060. 10.1128/Jb.00204-08 18375553 PMC2395044

[bib75] Onaka H., Mori Y., Igarashi Y., Furumai T. (2011). Mycolic acid-containing bacteria induce natural-product biosynthesis in *Streptomyces* species. Applied and Environmental Microbiology, 77, 400–406. 10.1128/AEM.01337-10 21097597 PMC3020563

[bib76] Onaka H., Ozaki T., Mori Y., Izawa M., Hayashi S., Asamizu S. (2015). Mycolic acid-containing bacteria activate heterologous secondary metabolite expression in *Streptomyces lividans*. Journal of Antibiotics, 68, 594–597. 10.1038/ja.2015.31 25829201

[bib77] Park H. B., Park J. S., Lee S. I., Shin B., Oh D. C., Kwon H. C. (2017). Gordonic acid, a polyketide glycoside derived from bacterial coculture of *Streptomyces* and *Gordonia* species. Journal of Natural Products, 80, 2542–2546. 10.1021/acs.jnatprod.7b00293 28845982

[bib78] Patin N. V., Floros D. J., Hughes C. C., Dorrestein P. C., Jensen P. R. (2018). The role of inter-species interactions in *Salinispora* specialized metabolism. Microbiology, 164, 946–955. 10.1099/mic.0.000679 29877785 PMC6152374

[bib79] Pel H. J., de Winde J. H., Archer D. B., Dyer P. S., Hofmann G., Schaap P. J., Turner G., de Vries R. P., Albang R., Albermann K., Andersen M. R., Bendtsen J. D., Benen J. A., van den Berg M., Breestraat S., Caddick M. X., Contreras R., Cornell M., Coutinho P. M., Stam H. (2007). Genome sequencing and analysis of the versatile cell factory *Aspergillus niger* CBS 513.88. Nature Biotechnology, 25, 221–231. 10.1038/nbt1282 17259976

[bib80] Pendleton J. N., Gorman S. P., Gilmore B. F. (2013). Clinical relevance of the ESKAPE pathogens. Expert Reviews of Anti-infective Therapy, 11, 297–308. 10.1586/Eri.13.12 23458769

[bib81] Perez J., Munoz-Dorado J., Brana A. F., Shimkets L. J., Sevillano L., Santamaria R. I. (2011). *Myxococcus xanthus* induces actinorhodin overproduction and aerial mycelium formation by *Streptomyces coelicolor*. Microbial Biotechnology, 4, 175–183. 10.1111/j.1751-7915.2010.00208.x 21342463 PMC3818858

[bib82] Pettit R. K. (2011). Small-molecule elicitation of microbial secondary metabolites. Microbial Biotechnology, 4, 471–478. 10.1111/j.1751-7915.2010.00196.x 21375710 PMC3815259

[bib83] Procopio R. E., Silva I. R., Martins M. K., Azevedo J. L., Araujo J. M. (2012). Antibiotics produced by *Streptomyces*. Brazilian Journal of Infectious Diseases, 16, 466–471. 10.1016/j.bjid.2012.08.014 22975171

[bib84] Quillet L., Barray S., Labedan B., Petit F., Guespin-Michel J. (1995). The gene encoding the β-1,4-endoglucanase (CelA) from *Myxococcus xanthus*: Evidence for independent acquisition by horizontal transfer of binding and catalytic domains from actinomycetes. Gene, 158, 23–29. 10.1016/0378-1119(95)00091-j 7789807

[bib85] Rateb M. E., Hallyburton I., Houssen W. E., Bull A. T., Goodfellow M., Santhanam R., Jaspars M., Ebel R. (2013). Induction of diverse secondary metabolites in *Aspergillus fumigatus* by microbial co-culture. RSC Advances, 3, 14444–14450. 10.1039/c3ra42378f

[bib86] Reen F. J., Romano S., Dobson A. D. W., O'Gara F. (2015). The sound of silence: Activating silent biosynthetic gene clusters in marine microorganisms. Marine Drugs, 13, 4754–4783. 10.3390/md13084754 26264003 PMC4557003

[bib87] Rice L. B. (2008). Federal funding for the study of antimicrobial resistance in nosocomial pathogens: No ESKAPE. Journal of Infectious Diseases, 197, 1079–1081. 10.1086/533452 18419525

[bib88] Romano S., Jackson S. A., Patry S., Dobson A. D. W. (2018). Extending the “one strain many compounds” (OSMAC) principle to marine microorganisms. Marine Drugs, 16, 244. 10.3390/md16070244 PMC6070831 30041461

[bib89] Saito S., Kato W., Ikeda H., Katsuyama Y., Ohnishi Y., Imoto M. (2020). Discovery of “heat shock metabolites” produced by thermotolerant actinomycetes in high-temperature culture. Journal of Antibiotics, 73, 203–210. 10.1038/s41429-020-0279-4 32015464

[bib90] Sanchez S., Chavez A., Forero A., Garcia-Huante Y., Romero A., Sanchez M., Rocha D., Sanchez B., Avalos M., Guzman-Trampe S., Rodriguez-Sanoja R., Langley E., Ruiz B. (2010). Carbon source regulation of antibiotic production. Journal of Antibiotics, 63, 442–459. 10.1038/ja.2010.78 20664603

[bib91] Sankaran L., Pogell B. M. (1975). Biosynthesis of puromycin in *Streptomyces alboniger*: Regulation and properties of *O*-demethylpuromycin *O*-methyltransferase. Antimicrobial Agents and Chemotherapy, 8, 721–732. 10.1128/aac.8.6.721 1211926 PMC429454

[bib92] Schaberle T. F., Orland A., Konig G. M. (2014). Enhanced production of undecylprodigiosin in *Streptomyces coelicolor* by co-cultivation with the corallopyronin A-producing myxobacterium, *Corallococcus coralloides*. Biotechnology Letters, 36, 641–648. 10.1007/s10529-013-1406-0 24249103

[bib93] Schroeckh V., Scherlach K., Nutzmann H. W., Shelest E., Schmidt-Heck W., Schuemann J., Martin K., Hertweck C., Brakhage A. A. (2009). Intimate bacterial–fungal interaction triggers biosynthesis of archetypal polyketides in *Aspergillus nidulans*. Proceedings of the National Academy of Sciences of the USA, 106, 14558–14563. 10.1073/pnas.0901870106 19666480 PMC2732885

[bib94] Shin D., Byun W. S., Moon K., Kwon Y., Bae M., Um S., Lee S. K., Oh D. C. (2018). Coculture of marine *Streptomyces* sp. with *Bacillus* sp. produces a new piperazic acid-bearing cyclic peptide. Frontiers in Chemistry, 6, 498. 10.3389/fchem.2018.00498 30406080 PMC6201156

[bib95] Slattery M., Rajbhandari I., Wesson K. (2001). Competition-mediated antibiotic induction in the marine bacterium *Streptomyces tenjimariensis*. Microbial Ecology, 41, 90–96. 12032613 10.1007/s002480000084

[bib96] Straight P. D., Fischbach M. A., Walsh C. T., Rudner D. Z., Kolter R. (2007). A singular enzymatic megacomplex from *Bacillus subtilis*. Proceedings of the National Academy of Sciences of the USA, 104, 305–310. 10.1073/pnas.0609073103 17190806 PMC1765455

[bib97] Stroe M. C., Netzker T., Scherlach K., Kruger T., Hertweck C., Valiante V., Brakhage A. A. (2020). Targeted induction of a silent fungal gene cluster encoding the bacteria-specific germination inhibitor fumigermin. eLife, 9, e52541. 10.7554/eLife.52541 32083553 PMC7034978

[bib98] Sugiyama R., Nishimura S., Ozaki T., Asamizu S., Onaka H., Kakeya H. (2015). 5-Alkyl-1,2,3,4-tetrahydroquinolines, new membrane-interacting lipophilic metabolites produced by combined culture of *Streptomyces nigrescens* and *Tsukamurella pulmonis*. Organic Letters, 17, 1918–1921. 10.1021/acs.orglett.5b00607 25826296

[bib99] Sugiyama R., Nishimura S., Ozaki T., Asamizu S., Onaka H., Kakeya H. (2016). Discovery and total synthesis of streptoaminals: Antimicrobial [5,5]-spirohemiaminals from the combined-culture of *Streptomyces nigrescens* and *Tsukamurella pulmonis*. Angewandte Chemie, International Edition, 55, 10278–10282. 10.1002/anie.201604126 27459894

[bib100] Sung A. A., Gromek S. M., Balunas M. J. (2017). Upregulation and identification of antibiotic activity of a marine-derived *Streptomyces* sp. via co-cultures with human pathogens. Marine Drugs, 15, 250. 10.3390/md15080250 PMC5577605 28800088

[bib101] Tacconelli E., Carrara E., Savoldi A., Harbarth S., Mendelson M., Monnet D. L., Pulcini C., Kahlmeter G., Kluytmans J., Carmeli Y., Ouellette M., Outterson K., Patel J., Cavaleri M., Cox E. M., Houchens C. R., Grayson M. L., Hansen P., Singh N., … WHO Pathogens Priority List Working Group. (2018). Discovery, research, and development of new antibiotics: The WHO priority list of antibiotic-resistant bacteria and tuberculosis. Lancet Infectious Diseases, 18, 318–327. 10.1016/S1473-3099(17)30753-3 29276051

[bib102] Tan Z. Q., Leow H. Y., Lee D. C. W., Karisnan K., Song A. A. L., Mai C. W., Yap W. S., Lim S. H. E., Lai K. S. (2019). Co-culture systems for the production of secondary metabolites: Current and future prospects. The Open Biotechnology Journal, 13, 18–26.

[bib103] Tanaka Y., Hosaka T., Ochi K. (2010). Rare earth elements activate the secondary metabolite-biosynthetic gene clusters in *Streptomyces coelicolor* A3(2). Journal of Antibiotics, 63, 477–481. 10.1038/ja.2010.53 20551989

[bib104] Traxler M. F., Seyedsayamdost M. R., Clardy J., Kolter R. (2012). Interspecies modulation of bacterial development through iron competition and siderophore piracy. Molecular Microbiology, 86, 628–644. 10.1111/mmi.12008 22931126 PMC3481010

[bib105] Traxler M. F., Watrous J. D., Alexandrov T., Dorrestein P. C., Kolter R. (2013). Interspecies interactions stimulate diversification of the *Streptomyces coelicolor* secreted metabolome. mBio, 4, e00459–13. 10.1128/mBio.00459-13 23963177 PMC3747584

[bib106] Ueda K., Kawai S., Ogawa H., Kiyama A., Kubota T., Kawanobe H., Beppu T. (2000). Wide distribution of interspecific stimulatory events on antibiotic production and sporulation among *Streptomyces* species. Journal of Antibiotics, 53, 979–982. 10.7164/antibiotics.53.979 11099234

[bib107] van Bergeijk D. A., Terlouw B. R., Medema M. H., van Wezel G. P. (2020). Ecology and genomics of actinobacteria: New concepts for natural product discovery. Nature Reviews Microbiology, 18, 546–558. 10.1038/s41579-020-0379-y 32483324

[bib108] Vargas-Bautista C., Rahlwes K., Straight P. (2014). Bacterial competition reveals differential regulation of the pks genes by *Bacillus subtilis*. Journal of Bacteriology, 196, 717–728. 10.1128/JB.01022-13 24187085 PMC3911183

[bib109] Ventola C. L. (2015). The antibiotic resistance crisis: Part 1: Causes and threats. Pharmacy and Therapeutics, 40, 277–283. 25859123 PMC4378521

[bib110] Wakefield J., Hassan H. M., Jaspars M., Ebel R., Rateb M. E. (2017). Dual induction of new microbial secondary metabolites by fungal bacterial co-cultivation. Frontiers in Microbiology, 8, 1284. 10.3389/fmicb.2017.01284 28744271 PMC5504103

[bib111] Wang Y., Wang L., Zhuang Y., Kong F., Zhang C., Zhu W. (2014). Phenolic polyketides from the co-cultivation of marine-derived *Penicillium* sp. WC-29-5 and *Streptomyces fradiae* 007. Marine Drugs, 12, 2079–2088. 10.3390/md12042079 24714124 PMC4012438

[bib112] Wortman J. R., Gilsenan J. M., Joardar V., Deegan J., Clutterbuck J., Andersen M. R., Archer D., Bencina M., Braus G., Coutinho P., von Dohren H., Doonan J., Driessen A. J., Durek P., Espeso E., Fekete E., Flipphi M., Estrada C. G., Geysens S., Turner G. (2009). The 2008 update of the *Aspergillus nidulans* genome annotation: A community effort. Fungal Genetics and Biology, 46(Suppl. 1), S2–S13. 10.1016/j.fgb.2008.12.003 19146970 PMC2826280

[bib113] Wu C., Zacchetti B., Ram A. F., van Wezel G. P., Claessen D., Hae Choi Y. (2015). Expanding the chemical space for natural products by *Aspergillus*–*Streptomyces* co-cultivation and biotransformation. Scientific Reports, 5, 10868. 10.1038/srep10868 26040782 PMC4455117

[bib114] Wu M. H., Huang S. B., Lee G. B. (2010). Microfluidic cell culture systems for drug research. Lab on a Chip, 10, 939–956. 10.1039/b921695b 20358102

[bib115] Yamada Y., Sugamura K., Kondo K., Yanagimoto M., Okada H. (1987). The structure of inducing factors for virginiamycin production in *Streptomyces virginiae*. Journal of Antibiotics, 40, 496–504. 10.7164/antibiotics.40.496 3108224

[bib116] Yamanaka K., Oikawa H., Ogawa H. O., Hosono K., Shinmachi F., Takano H., Sakuda S., Beppu T., Ueda K. (2005). Desferrioxamine E produced by *Streptomyces griseus* stimulates growth and development of *Streptomyces tanashiensis*. Microbiology, 151, 2899–2905. 10.1099/mic.0.28139-0 16151202

[bib117] Yu L., Ding W., Ma Z. (2016). Induced production of cytochalasans in co-culture of marine fungus *Aspergillus flavipes* and actinomycete *Streptomyces* sp. Natural Product Research, 30, 1718–1723. 10.1080/14786419.2015.1136910 26783945

[bib118] Yu L., Hu Z., Ma Z. (2015). Production of bioactive tryptamine derivatives by co-culture of marine *Streptomyces* with *Bacillus mycoides*. Natural Product Research, 29, 2087–2091. 10.1080/14786419.2015.1005619 25643750

[bib119] Yu M., Li Y., Banakar S. P., Liu L., Shao C., Li Z., Wang C. (2019). New metabolites from the co-culture of marine-derived actinomycete *Streptomyces rochei* MB037 and fungus *Rhinocladiella similis* 35. Frontiers in Microbiology, 10, 915. 10.3389/fmicb.2019.00915 31134000 PMC6514141

[bib120] Zhang M. M., Wong F. T., Wang Y., Luo S., Lim Y. H., Heng E., Yeo W. L., Cobb R. E., Enghiad B., Ang E. L., Zhao H. (2017). CRISPR–Cas9 strategy for activation of silent *Streptomyces* biosynthetic gene clusters. Nature Chemical Biology, 13, 607–609. 10.1038/nchembio.2341 PMC5634907 28398287

[bib121] Zuck K. M., Shipley S., Newman D. J. (2011). Induced production of *N*-formyl alkaloids from *Aspergillus fumigatus* by co-culture with *Streptomyces peucetius*. Journal of Natural Products, 74, 1653–1657. 10.1021/np200255f 21667925

